# Bacterial co-infections and antibiotic resistance in patients with COVID-19

**DOI:** 10.3205/dgkh000370

**Published:** 2020-12-17

**Authors:** Hassan Mahmoudi

**Affiliations:** 1 Department of Microbiology, Faculty of Medicine, Hamadan University of Medical Sciences, Hamadan, Iran; 2 Department of Laboratory Medicine, Ayatollah Alimoradiyan Hospital, Nahavand, Hamadan, Iran

**Keywords:** COVID-19, bacterial co-infections, antibiotic resistance, viral respiratory infections, SARS-CoV-2

## Abstract

**Background:** Bacterial co-infections are frequently identified in viral respiratory infections and are significant reasons for morbidity and mortality. Information on the prevalence of bacterial co-infection in patients infected with severe acute respiratory syndrome coronavirus 2 (SARS-CoV-2) is lacking. The purpose of this study was to determine the prevalence of bacterial infections and antibiotic resistance in patients with coronavirus disease (COVID-19).

**Methods:** In a cross-sectional study, blood culture (BC) and endotracheal aspirate (ETA) were obtained from COVID-19 patients (RT-PCR positive for SARS-CoV-2). The bacterial isolates were confirmed by the standard microbiological methods. Antibiotic resistance was determined using the disk diffusion method.

**Results:** Among these 340 patients with COVID-19, a total of 43 (12.46%) patients had secondary bacterial infections. The most common bacteria isolated through ETA and BC included Klebsiella species 11 (25.59%), methicillin-sensitive *Staphylococcus aureus* (MSSA) 9 (20.93%), *Escherichia coli* 7 (16.28%), methicillin-resistant *Staphylococcus aureus* (MRSA) 6 (13.95%), *Enterobacter species* 5 (11.63%), *Streptococcus pneumoniae* 1 (2.32%), and *Pseudomonas aeruginosa* 4 (9.30%). The results showed that Enterobacteriaceae isolates from COVID-19 patients had the highest resistance to cotrimoxazole (74%), piperacillin (67.5%), ceftazidime (47.5%), and cefepime (42.5%). All isolates were susceptible to amikacin (100%). *S. aureus* isolates were susceptible to vancomycin (100%) and the rates of resistance to oxacillin, erythromycin and clindamycin were over (90%). *P. aeruginosa* was susceptible (90%) to imipenem.

**Conclusions:** Bacterial co-infection is relatively infrequent in hospitalized COVID-19 patients. According to the results, one of the causes of death of these patients could be a secondary infections.

## Zusammenfassung

**Hintergrund:** Bakterielle Co-Infektionen werden häufig bei viralen Atemwegsinfektionen identifiziert und sind wesentliche Gründe für Morbidität und Mortalität. Es fehlen Informationen über die Prävalenz bakterieller Co-Infektionen bei Patienten mit COVID-19.. Ziel dieser Studie war es, die Prävalenz von bakteriellen Infektionen und Antibiotikaresistenzen bei an COVID-19 erkrankten Patienten zu bestimmen.

**Methoden:** In einer Querschnittsstudie wurden Blutkulturen (BC) und endotracheale Aspirate (ETA) von COVID-19-Patienten (RT-PCR positiv für SARS-CoV-2) gewonnen. Die bakteriellen Isolate wurden mit den mikrobiologischen Standardmethoden bestätigt. Die Antibiotikaresistenz wurde im Plattendiffusionstest bestimmt.

**Ergebnisse:** Unter 340 Patienten mit COVID-19 hatten insgesamt 43 (12,46 %) Patienten bakterielle Sekundärinfektionen. Die häufigsten Bakterien, die durch ETA und BC isoliert wurden, waren Klebsiella-Spezies 11 (25,59%), Methicillin-sensitiver *Staphylococcus aureus* (MSSA) 9 (20,93%) und *Escherichia coli* 7 (16. 28%), Methicillin-resistenter *Staphylococcus aureus* (MRSA) 6 (13,95%), und Enterobacter-Arten 5 (11,63%) sowie *Streptococcus pneumoniae* 1 (2,32%) und *Pseudomonas aeruginosa* 4 (9,30%). Die Ergebnisse zeigten, dass Enterobacteriaceae-Isolate von COVID-19-Patienten die höchste Resistenz gegen Cotrimoxazol (74%), Piperacillin (67,5%), Ceftazidim (47,5%) und Cefepime (42,5%) aufwiesen. Alle Isolate waren empfindlich gegenüber Amikacin (100%). *S. aureus*-Isolate waren empfindlich gegenüber Vancomycin (100%) und die Resistenzraten gegenüber Oxacillin, Erythromycin und Clindamycin waren über 90%. *P. aeruginosa* war empfindlich (90%) gegenüber Imipenem.

**Schlussfolgerungen:** Bakterielle Co-Infektionen sind bei hospitalisierten COVID-19-Patienten relativ selten. Nach den Ergebnissen könnte eine der Todesursachen dieser Patienten eine Sekundärinfektion sein.

## Introduction

Bacterial co-infections are frequently determined in viral respiratory tract infections, such as influenza, and are a significant cause of morbidity and mortality. Thus, timely diagnosis and antibacterial treatment are necessary [1], [2], [3] The frequency, incidence and features of bacterial co-infection in patients infected with (SARS-CoV-2) are not clear; in these critical circumstances, this is a crucial knowledge gap [4], [5], [6], [7] Although antibiotics are ineffective treatment of COVID-19, physicians prescribed them for patients with suspected or documented COVID-19 for a variety of reasons [8]. In terms of the mortality rate of patients with bacterial supra-infection during influenza pandemics, several guidelines support the usage of empirical antibiotic therapy for COVID-19 patients [8]. It is difficult to rule out bacterial co-infection on presentation, but also the possibility of bacterial secondary infection during the course of the disease. Nevertheless, this approach increases concerns about antibiotic overuse and subsequent detrimental consequences related to bacterial resistance. Given a rise in mortality in patients with bacterial supra-infection during influenza pandemics, several guidelines supporting the application of empirical antibiotics for patients with severe COVID-19 have been developed [8]. Given the fact that Covid-19 patients can have a bacterial co-infection and bearing in mind the action of the pathogens, it is critical to treat Covid-19 patients responsibly in terms of antibiotics in order to minimize the negative effects of overuse [6]. Furthermore, the rate of bacterial co-infection in Covid-19 patients could have an important influence on refining empirical antibiotic management guidelines for patients with COVID-19. The purpose of this study was to identify the prevalence of bacterial co-infection in COVID-19 patients.

## Methods

### Study design

Sampling for this cross-sectional study was done at Nahavand Hospitals, Hamedan, Iran. The study was performed from February 17, 2020 to October 20, 2020. Eligibility for participating in the study was determined by criteria itemized in a questionnaire completed by each patient. Initial laboratory investigations included complete blood count (CBC), erythrocyte sedimentation rate (ESR), arterial blood gas (ABG), lactate dehydrogenase (LDH), and C-reactive protein (CRP) tests. Serial monitoring of the laboratory profile was performed according to the clinical progress of the individual patient. All patients were laboratory-confirmed positive for SARS-CoV-2 by use of quantitative RT-PCR (qRT-PCR) on throat-swab samples. We reviewed the clinical laboratory findings for all the COVID-19 patients. All information was obtained and recorded in a customized data collection form. 

### Culture and isolation of bacteria

BC and ETA cultures were obtained from COVID-19 patients. In order to differentiate microorganisms, swabs and blood were cultured on blood agar and MacConkey agar plates and incubated at 37°C for 18–24 hours. Identification of the isolated bacteria was performed using standard microbiological methods [9]. 

### Antibacterial susceptibility

For all isolated strains, antibacterial susceptibility was tested using the standard Kirby-Bauer disk-diffusion method on Mueller Hinton agar (Merk Co., Germany) in accordance with the clinical and laboratory standards institute guidelines (CLSI; 2019, M100-S29) using gentamicin (10 µg), vancomycin (30 µg), trimethoprim/sulfamethoxazole (25 µg), amikacin (30 µg), tobramycin (10 µg), cephalotin (30 µg), norfloxacin (5 µg), and ceftizoxim (30 µg) disks (Mast Co.UK) ([10], [11]).

## Results

**Figure 1:**
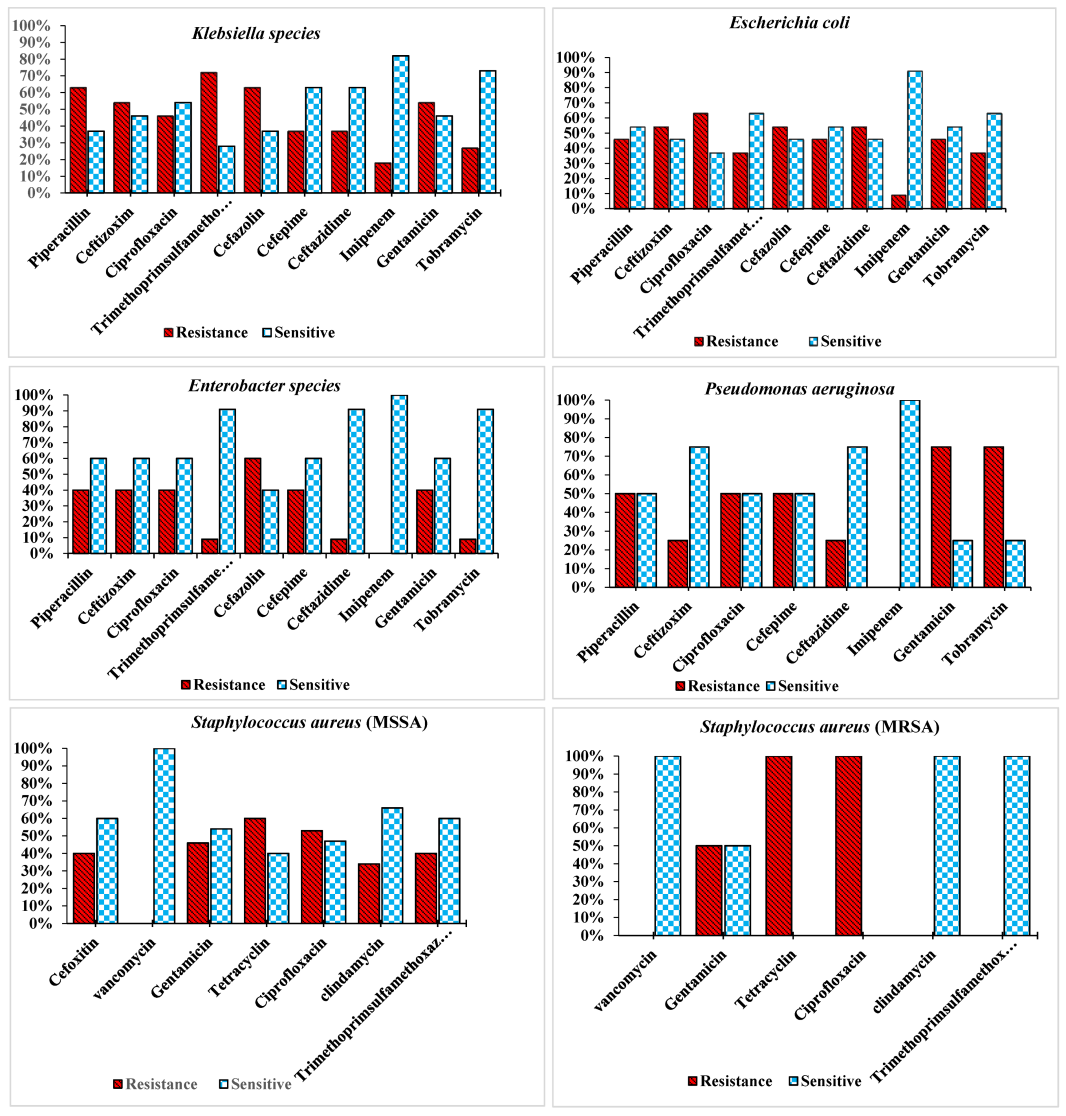
Antimicrobial susceptibility patterns of bacterial strains isolated from COVID-19 patients

A total of 43 positive cultures (12.46%) of the blood and endotracheal aspirate samples were obtained. The most common bacteria isolated from endotracheal aspirate and blood cultures included Klebsiella species 11 (25.59%), *S. aureus* (MSSA) 9 (20.93%), *E. coli* 8 (18.6%), *S. aureus* (MRSA), 6 (13.95%), and Enterobacter species 5 (11.63%), as well as confirmed presence of *P. aeruginosa* 4 (9.30%) (Table 1 (Tab. 1)). The sensitivity patterns to tested antibiotics are shown in Figure 1 (Fig. 1).

## Discussion

COVID-19, a viral pneumonia that is now a pandemic, is considered a novel public health concern. Recent studies show that 2019-nCoV originated from an animal source and later adapted to other variants as it crossed the species barrier to ultimately infect humans. It is well established that seasonal viral respiratory tract infections are related to increased risk of bacterial co-infection. In previous influenza pandemics, bacterial co-infections have been a major cause of mortality [12]. We aimed to evaluate the burden of co-infections in patients with COVID-19. Among these 340 patients with COVID-19, secondary bacterial infections occurred in a total of 43 (12.46%) patients. The most common bacteria isolated from endotracheal aspirate and blood cultures included *Klebsiella* species, *S. aureus* (MSSA), and *E. coli*, *S. aureus* (MRSA), and *Enterobacter* species, and the presence of *S. pneumoniae* and *P. aeruginosa*. Moreover, hospital admissions increase the risk of health-care related infections and the transmission of multidrug-resistant organisms, which in turn lead to increased use of antibiotics. A recent study in intensive care units (ICU) in 88 countries showed that although only 54% of patients had suspected or confirmed bacterial co-infection, 70% of them had received at least one antibiotic either as treatment or as antimicrobial prophylaxis [13]. At Montefiore medical center (New York City), Amy Norton [14] reported this earlier in the pandemic: Of more than 5,800 COVID-19 patients hospitalized from March through May, 2020, 71% received at least one antibiotic drug dose [14]. Two studies published on hospitalized COVID-19 patients determined that while 72% of patients received antibiotics, only 8% confirmed bacterial supra-infection or fungal co-infections [13][14]. Moreover, in the study by Sharifipour et al. [10], an evaluation of bacterial co-infections of the respiratory tract in COVID-19 patients admitted to the ICU also showed secondary infections with *Acinetobacter baumannii* and two *S. aureus* strains. Yang et al. [15] reported that hospital-acquired infections were prominent in 13.5% of patients, including one (2%) patient who had pulmonary and blood-stream infection with *K. pneumoniae*. Other microorganisms recognized from respiratory tract secretions in five (10%) patients included Aspergillus flavus, A fumigatus, extended spectrum β-Lactamase (ESBL)-positive *K. pneumonia*, ESBL-positive *P. aeruginosa*, and ESBL-negative *Serratia marcescens*, with each microorganism found in one patient each. *Candida albicans* was detected in the urine culture of one (2%) patient. In another study, Xavier Lescure et al. [16] identified two pathogens: antibiotic-susceptible *A. baumannii* and *A. flavus*. Zhou et al. [17] showed that half of the patients with COVID-19 developed sepsis. In the current study, we found that in 12.5% patients, secondary bacterial infections occurred. Guo et al. [18] reported that some patients, particularly severely ills ones, had co-infections with bacteria. The usual bacterial cultures from patients with secondary infections identified *A. baumannii* and *K. pneumoniae*. Compared with *K. pneumoniae*,* A. baumannii* was more highly resistant to antibiotics. Cucchiari [19] and Xing [20] in two separate studies reported various incidence rates of simultaneous bacterial infection in COVID-19. Xing et al. [19] found the most common respiratory pathogens in COVID-19 patients to be *Mycoplasma pneumoniae* (23.33%) and *Legionella pneumophila* (20%). Xing et al. [19] in two separate studies reported 1 to 10% of COVID-19 patients to contract secondary bacterial infections. In a systematic review, Lansbury et al. [12] reported that 7% of hospitalized COVID-19 patients had a bacterial co-infection. The most common bacteria were *M. pneumonia*, *P. aeruginosa,* and *Haemophilus influenza*. Toombs et al. [21] found that two COVID-19 patients (0.4%) were co-infected with *S. pneumoniae*, as determined by blood culture positivity upon hospitalization in the United Kingdom. These results support the hypothesis of secondary infection in COVID 19 patients. According to information from studies of antibiotic usage in treating COVID-19 patients, an average 70% of patients receive antibiotics. Nevertheless, extreme caution should be used, given that inappropriate usage or overuse of antibiotics is known to be an important driver of the emergence of antimicrobial resistance. This is why significant efforts against antimicrobial resistance revolve around reducing inappropriate or overuse of antibiotics [22]. Excessive use of antimicrobial soaps and disinfectants by hospital staff will have become more common over the last few months. If these products based on chlorhexidine digluconate or quaternary ammonium compounds, this too can lead to antibiotic resistance. The present study found that COVID-19 patients with secondary bacterial infections were highly resistant to common antibiotics in the isolated bacteria. The results showed that *Enterobacteriaceae* isolates from COVID-19 patients had the highest resistance to cotrimoxazole (74%), piperacillin (67.5%), ceftazidime (47.5%), and cefepime (42.5%); all isolates were susceptible to amikacin (100%). *S. aureus* isolates were susceptible to vancomycin (100%), but the rates of resistance to oxacillin, erythromycin and clindamycin were over (90%); furthermore, *P. aeruginosa* spp. was susceptible (90%) to imipenem. Another possible threat is the extensive application of biocidal agents for personal and environmental disinfection in non-healthcare settings. A low level of exposure to biocidal agents can select for drug resistant strains and increase the risk of cross-resistance to different antibiotics, mainly those that treat Gram-negative bacteria [13]. In recent months, less attention has been paid to nosocomial infections and opportunistic microorganisms, which could be due to the outbreak of COVID-19, its consequent long-term hospitalization of patients, and high workload on the healthcare personnel. 

Nevertheless, our findings were based on a limited number of observational studies. Further well-designed studies with larger sample sizes are necessary to increase our knowledge of the prevalence and risk of COVID-19 bacterial co-infection, as well as the influence of co-infection on the clinical outcomes of COVID-19 patients.

## Conclusions

The current report highlights the need to consider co-infection of SARS-CoV-2 with other pathogens to optimize treatment. After obtaining more data regarding co-infection with SARS-CoV-2, empirical antimicrobial agents in suspected COVID-19 cases can be suggested.

## Abbreviation

**SARS-CoV-2:** Severe acute respiratory syndrome coronavirus 2

**COVID-19:** coronavirus disease

**BC:** Blood culture

**ETA:** Endotracheal aspirate

**MSSA:** Methicillin sensitive *Staphylococcus aureus*

**MRSA:** Methicillin resistance *Staphylococcus aureus*

**ESBL:** Extended spectrum β-Lactamase 

**CBC:** Complete blood count 

**ESR:** Erythrocyte sedimentation rate 

**ABG:** Arterial blood gas 

**LDH:** lactate dehydrogenase 

**CRP:** C-reactive protein

**QRT-PCR:** Quantitative RT-PCR

## Notes

### Competing interests

The author declares that he has no competing interests.

### Acknowledgements

I would like to thank all members of laboratory medicine of Ayatollah Alimoradiyan Hospital, Nahavand, Hamadan, Iran. 

### Funding

This research was supported by Vice Chancellor for Research & Technology of Hamadan University of Medical Sciences, Hamadan, Iran. 

### Authors’ contributions

All stages of this study were performed by HM.

### Ethics approval and consent to participate

This study has been approved by the Hamadan University of Medical Sciences, Iran, ethics code IR.UMSHA.REC.1399.095.

## Figures and Tables

**Table 1:**
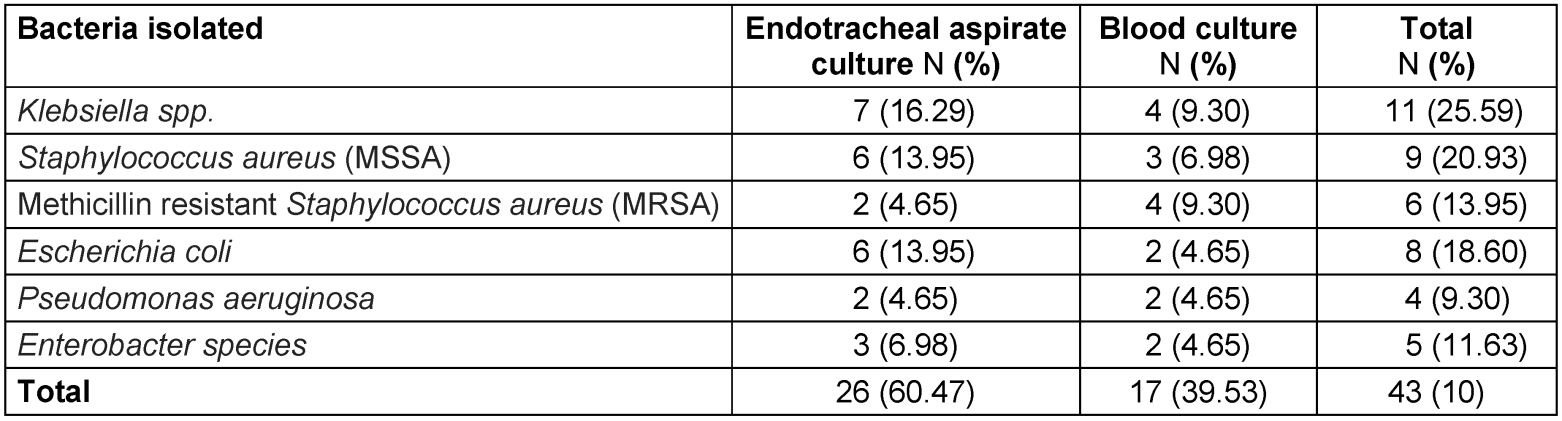
Occurrence and frequency of bacterial species isolated from COVID-19 patients
